# Flexible Two-Dimensional Square-Grid Coordination Polymers: Structures and Functions

**DOI:** 10.3390/ijms11103803

**Published:** 2010-09-30

**Authors:** Hiroshi Kajiro, Atsushi Kondo, Katsumi Kaneko, Hirofumi Kanoh

**Affiliations:** 1 Nippon Steel Corporation, 20-1Shintomi, Futtsu, Chiba 293-8511, Japan; 2 Department of Chemistry, Graduate School of Science, Chiba University, Yayoi, Inage, Chiba 263-8522, Japan; E-Mail: kanoh@pchem2.s.chiba-u.ac.jp; 3 Collaborative Innovation Center for Nanotech FIBER (nanoFIC), Shinshu University, 3-15-1 Tokida, Ueda, Nagano 386-8567, Japan; E-Mail: kondoa@cc.tuat.ac.jp; 4 Research Center for Exotic Nanocarbons, Shinshu University, Wakasato 4-17-1, Nagano-city 380-8553, Japan; E-Mail: kkaneko@shinshu-u.ac.jp

**Keywords:** porous coordination polymer (PCP), metal-organic framework (MOF), gas adsorption, gas separation, structural transformation, gate phenomena, elastic layer-structure, clathrate formation

## Abstract

Coordination polymers (CPs) or metal-organic frameworks (MOFs) have attracted considerable attention because of the tunable diversity of structures and functions. A 4,4′-bipyridine molecule, which is a simple, linear, exobidentate, and rigid ligand molecule, can construct two-dimensional (2D) square grid type CPs. Only the 2D-CPs with appropriate metal cations and counter anions exhibit flexibility and adsorb gas with a gate mechanism and these 2D-CPs are called elastic layer-structured metal-organic frameworks (ELMs). Such a unique property can make it possible to overcome the dilemma of strong adsorption and easy desorption, which is one of the ideal properties for practical adsorbents.

## 1. Introduction

Organic synthetic chemistry has enabled us to develop various kinds of elegant synthetic methods such as C-C bond formations, condensation reactions, and functional group transformations, and has realized the synthesis of extremely complicated organic functional molecules [1–3]. On the other hand, supramolecule chemistry shows us that the creation of complicated structures and functions may be possible even by mere mixing of components (self-organization) when we utilize weak interactions such as coordination bond, hydrogen bonding, and π-π interaction [4–8].

Coordination polymers (CPs) or metal-organic frameworks (MOFs) which are synthesized from exo-multidentate ligands and metal cations through the self-organization process have attracted considerable attention because of their diversity of structures and functions with the appropriate tunability [9–23]. In particular, porous coordination polymers (PCPs) or porous MOFs are considered as a promising candidate for a new class of adsorbent [24–26], separation material [27–30], catalyst [31–41], and sensors [42], because of their high sorption capacities and molecular recognition abilities by excellent tunability of the pore structure [11,43,44].

One of the characteristics of PCPs/MOFs is a softness derived from the weak interactions between counter ions and ligands, or ligands and ligands. Since traditional porous materials such as zeolite or activated carbon are ordinarily robust, adsorbed guest molecules are accommodated into the steadily constructed pores. On the other hand, some kinds of PCPs/MOFs show structural flexibility [45–62]. The flexible PCPs/MOFs interact with guest molecules, showing nonporous/porous structural transformations [63–66] or change the pore structures in response to external stimuli [67–70]. In the case of gas adsorption phenomena on robust traditional porous materials, the adsorbed amount tends to increase gradually with the increment of gas pressure. However, flexible PCPs/MOFs, in some cases, show non-linear responses between the adsorbed amount and the gas pressure. Although such an interesting phenomenon has been extensively studied in the case of crystals of small organic molecules or a discrete complex [71–78], the detailed mechanism of non-linear responses of PCPs/MOFs in gas adsorption is still unclear. In this review, we introduce the structures and functions of flexible two dimensional PCPs/MOFs, which are constructed with simple, rigid, and linear ligands, 4,4′-bipyridine (bpy), and are named “elastic layer-structured metal-organic frameworks (ELMs)”. We also discuss the advantages of flexible ELMs for practical applications.

## 2. Gas Adsorptivity of Elastic Layer-Structured Metal-Organic Frameworks (ELMs)

### 2.1. Discovery of Gate Phenomena of Coordination Polymer

**Figure 1:**
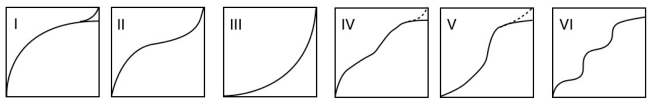
Six types of IUPAC adsorption isotherms: X-axis is relative pressure and Y-axis is adsorption amount. Typical traditional nanoporous materials are ordinarily classified into type I adsorption isotherm.

In 2001, Li and Kaneko reported interesting gas adsorption phenomena on a blue crystalline coordination polymer synthesized from bpy and Cu(BF_4_)_2_ showing sudden gas uptake at a definite gas pressure [63]. Porous and nonporous materials show that various gas adsorption isotherms depend on the surface properties, the pore diameter, and nature of the gas molecule. The adsorption isotherms for vapors are classified into six types by the IUPAC (Figure 1) [79].

**Figure 2:**
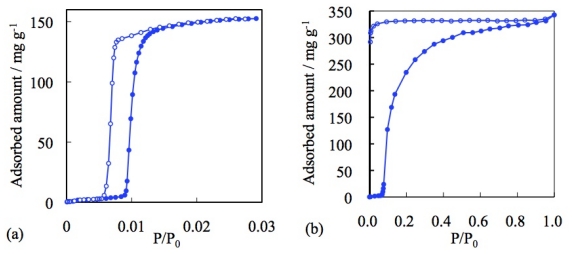

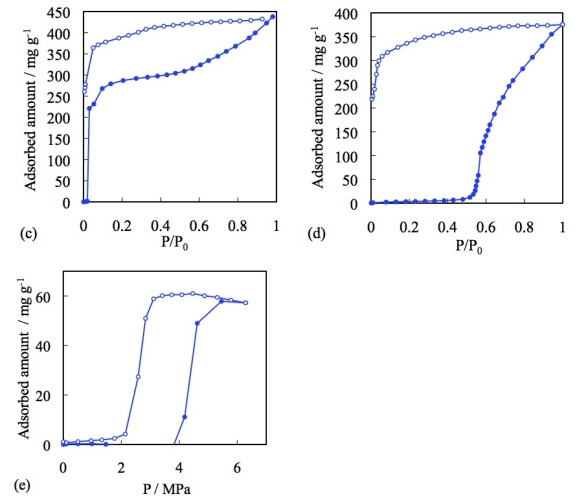
Gate phenomena of the “blue crystalline” CP/MOF with various gases: (**a**) CO_2_ at 273 K, (**b**) N_2_ at 77 K, (**c**) O_2_ at 77 K, (**d**) Ar at 77 K, and (**e**) CH_4_ at 303 K.

Despite the difference of the detail profile, all of the six types of adsorption isotherms show a gradual increase of the amount of gas adsorption dependent on gas pressure. Therefore, the nil gas adsorption in the low-pressure region and the sudden gas uptake profile of the “blue crystalline” CP/MOF cannot be classified by IUPAC categories. There were a number of reports on gas adsorption phenomena on PCPs/MOFs before the Li-Kaneko report in 2001. In the old example, although adsorption isotherms were not disclosed, Mori and Takamizawa reported the gas adsorption phenomena on copper complexes. Nevertheless, all of the PCPs/MOFs show traditional adsorption isotherms, which are classified by the six types of adsorption isotherms [80–93]. In other words, before the Li-Kaneko report, all of the gas adsorption phenomena on PCPs/MOFs were classified into representative physisorption by porous materials and resembled such properties, which were shown by traditional adsorbents. In this context, gate adsorption was an unprecedented phenomenon. Subsequent studies revealed that the “blue crystalline” CP/MOF shows the gate phenomena not only to CO_2_ but also N_2_, O_2_, and Ar [94,95]. It is noteworthy that the “blue crystalline” CP/MOF also shows a gate response to supercritical CH_4_ at 303 K that usually shows very small interaction to adsorbent (Figure 2) [96].

### 2.2. Structure of Two-Dimensional Layer-Stacking Coordination Polymer

**Figure 3:**
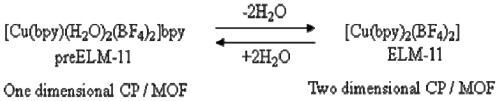
Interconversion of preELM-11 (**1**) and ELM-11.

The chemical formula of the “blue crystalline” CP is [Cu(bpy)(H_2_O)_2_(BF_4_)_2_]·bpy (**1**), and Hubberstey *et al*. firstly reported its structure [97]; in which a one-dimensional main structure that is composed of Cu^2+^-bpy is integrated into a three-dimensional structure through hydrogen bondings among coordinated H_2_O, bridging guest bpy molecules, and BF_4_^−^ anions. This CP shows the gate adsorption properties after heating *in vacuo* treatment; hence, the characteristic hydrogen bonding network was thought to play an important role in the gate phenomenon in the early stage of the study. In a detailed study by X-ray diffraction analysis, infrared spectroscopy (IR), EXAFS, and elemental analysis, it is revealed that the CP releases water molecules in a reversible fashion and changes its structure into a two-dimensional layer stacking-type architecture, and its chemical formula is [Cu(bpy)_2_(BF_4_)_2_] (Figure 3) [98]. This layered CP was revealed to be the real gate material and named an elastic layer-structured metal-organic framework (ELM-11). Therefore the hydrated complex **1** is named preELM-11.

**Figure 4:**
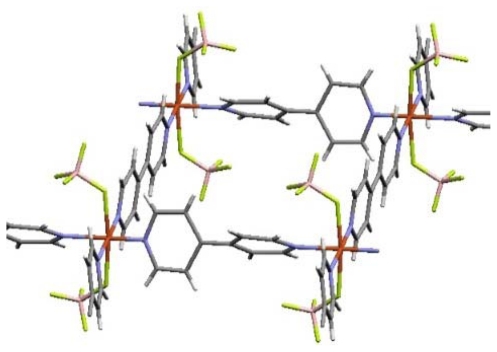
The local structure of ELM-11 (orange, Cu; gray, C; pale purple, N; pink, B; yellow green, F; white, H).

The Cu^2+^ ions are octahedrally coordinated by four bpy ligands at the equatorial positions to give two-dimensional, square grid sheets (Cu-Cu squares: 11.15 × 11.15 Å), while two BF_4_^−^ anions occupy the transaxial positions (Figure 4) [99]. Although the square grid motif constructed by linear bidentate ligand and Cu^2+^ is not uncommon, BF_4_^−^ anion, which has a weak coordination ability and coordinated structure, is relatively unique [100–106].

**Figure 5:**
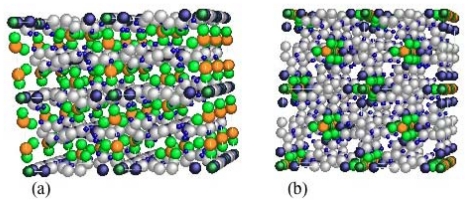
Layer stacking structure of ELM-11: (**a**) side view and (**b**) top view.

Although there are spaces for the inclusion of guest molecules in each square cavity, there is no effective pore in the stacked architecture because of the staggered stacking structure (Figure 5). The nil adsorption under the gate pressure can be understood from this structure, and Kaneko named this kind of CP a latent porous crystal, LPC [96]. Later the name of LPC was extended into a general name which covers family compounds, as given in this review.

**Figure 6:**
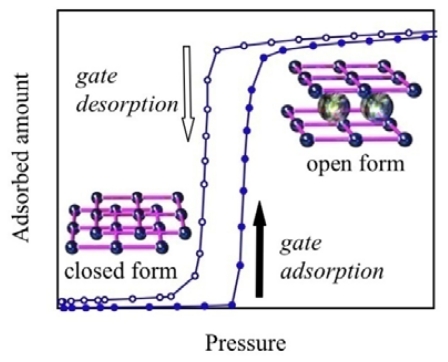
Schematic representation of the gate adsorption and transformation of ELM-11 between the closed and the open form.

**Figure 7:**
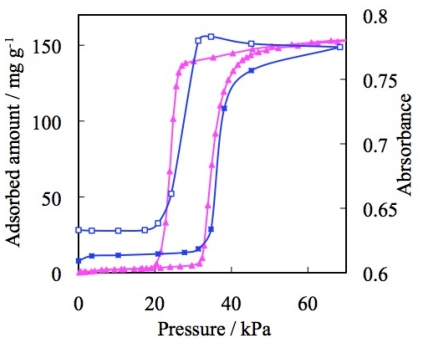
The correspondence of CO_2_ adsorption isotherm and IR spectral data: Carbon dioxide adsorption/desorption (pink) on ELM-11 and IR spectra (absorbance change of the peak (BF_4_^−^), blue) at 273 K.

**Figure 8:**
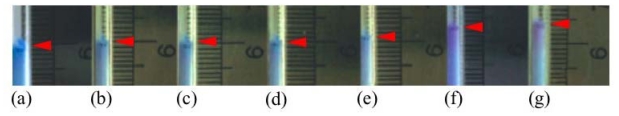
Volume change of ELM-11 accompanied by CO_2_ adsorption at 273 K: (**a**) before CO_2_ adsorption and after CO_2_ adsorption at (**b**) 6.66 kPa; (**c**) 13.3 kPa; (**d**) 26.7 kPa; (**e**) 34.7 kPa; (**f**) 45.3 kPa; (**g**) 101 kPa.

The unique gate phenomenon was clarified by detailed synchrotron radiation experiments on the CO_2_ adsorbed structure of the CP [99]. After CO_2_ adsorption, the inter-layer distance is increased by 1.20 Å (26%) from 4.58 Å to 5.78 Å, and the staggered stacking layers slide with each other, accompanying the rotation of the pyridine ring [107–109]. As a consequence, spaces for the accommodation of guest molecules are generated. Such structural change was also confirmed by infrared spectroscopy (IR); the peak at 1149 cm^−1^, which is assigned to the BF_4_^−^, immediately disappeared during CO_2_ adsorption, and a new peak appeared at 1170 cm^−1^ by way of compensation. Two isosbestic points at 1144 and 1156 cm^−1^ indicate that this phenomenon is a transformation between two states: The apohost and the CO_2_-CP clathrate (Figure 6) [96]. The IR spectral change and CO_2_ adsorption isotherm show relatively good correspondence (Figure 7). The gate gas adsorption and inter-layer expansion were accompanied with volume changes of powder crystalline ELM-11 (Figure 8). It is noteworthy that molecular expansion phenomena cause a macroscopic volume change irrespective of non negligible outer granular gaps.

In short, the gate phenomenon of ELM-11 is summarized as follows: (1) gate adsorption and desorption processes are ascribed to expansive, and shrinking modulation of the layer-stacking structure accompanied with gas molecule accommodation and effluence, respectively [55,110]; (2) the structural change consists of a two-state transformation between the apohost and guest-apohost clathration; (3) in a microscopic sense, the structural change is induced by the molecular movement generating the accommodation space, such as layer sliding, interlayer expansion, and pyridine ring rotation, caused by guest molecule accommodation and in the macroscopic sense, powder volume enhancement caused by external stimuli [111,112].

## 3. Elastic Layer-Structured Metal-Organic Frameworks (ELMs)

**Figure 9:**
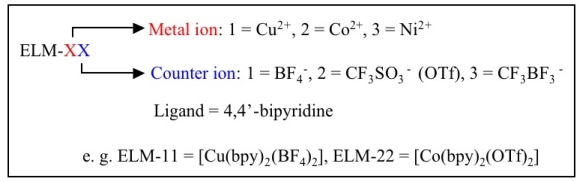
Nomenclature of ELMs.

Here a detailed expansion of the name of ELMs is given. We named the expansive/shrinking flexible CP/MOF as an ELM—an elastic layer-structured metal-organic framework. A series of isostructural ELMs with various metal ions, counter ions, and ligands were developed by our group. The metal and counter ions play an essential role in the structure and property and thereby the composition is added to ELMs for a specified ELM family, with the compositions of their components shown in Figure 9.

All the ELMs show gate adsorption/desorption behavior, and comparing the results of the investigation of the phenomena has revealed the roles of each component: metal ions, counter ions, and ligands.

### 3.1. Role of the Counter Ions

**Figure 10:**
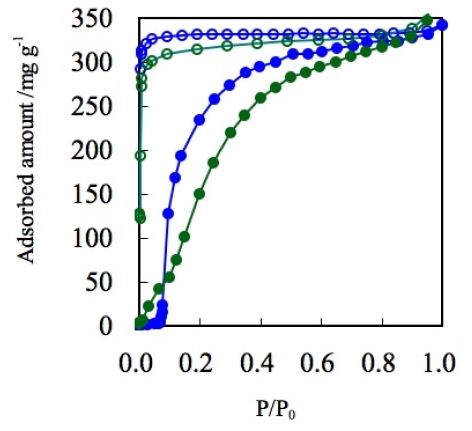
Adsorption isotherms of N_2_ on ELM-11 (Cu-BF_4_) (blue circles) and ELM-31 (Ni-BF_4_)(green circles) at 77 K. Solid and open symbols represent adsorption and desorption, respectively.

In the case of nitrogen gas adsorption at 77 K, BF_4_^−^ containing ELM-11 ([Cu(bpy)_2_(BF_4_)_2_]) and ELM-31 ([Ni(bpy)_2_(BF_4_)_2_]), both show that the upward convex profile in the adsorption isotherm and the maximum amount of adsorption are almost similar (Figure 10). Although the structure of [Ni(bpy)_2_(BF_4_)_2_] is still unclear because of the difficulty of single crystal synthesis, the composition of the components was confirmed by elemental analysis. The similarity of ELM-11 and ELM-31 was confirmed by IR, TG, and gas adsorption experiments using several gases, such as N_2_, CO_2_, and O_2_. Therefore, the structure of the Ni-CP is presupposed to a two-dimensional square grid in this article.

**Figure 11:**
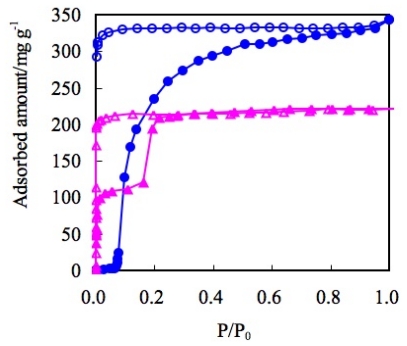
Adsorption isotherms of N_2_ on ELM-11 (Cu-BF_4_) (blue circles) and ELM-12 (Cu-OTf)(pink triangles) at 77 K. The solid and open symbols represent adsorption and desorption, respectively.

On the other hand, ELM-12 (Cu), containing trifluoromethanesulfonate (OTf) as a counter ion, shows a definite double step adsorption isotherm, while the rising profile is almost vertical (Figure 11) [67]. From the detailed structural study using synchrotron X-ray diffraction analysis, it was revealed that the ELM-12 has open micropores at the initial stage, and the first vertical adsorption step is assigned to micropore filling in the inherent micropores. This is in marked contrast to the nonporous nature of ELM-11, in spite of the common analogous fundamental two-dimensional layer structure. This difference is derived from only slight difference in stacking structure; the layers of ELM-11 are stacked in a complete staggered form, so that the bpy molecules cover the open space of metal-organic square grids in the neighboring layers. On the other hand, the layers of ELM-12 are also stacked in zigzag fashion, but the slipped degree is smaller than that of ELM-11 hence ELM-12 affords effective micropores to accommodate gases. In addition, the slightly larger inter-layer distance of ELM-12 compared to that of ELM-11 also help generate porosity at the initial stage. Furthermore, it is also revealed that the second adsorption step is derived from layer expansion phenomena accompanied with the gas-ELM clathrate generation in a way analogous to the gate phenomena of ELM-11.

**Figure 12:**
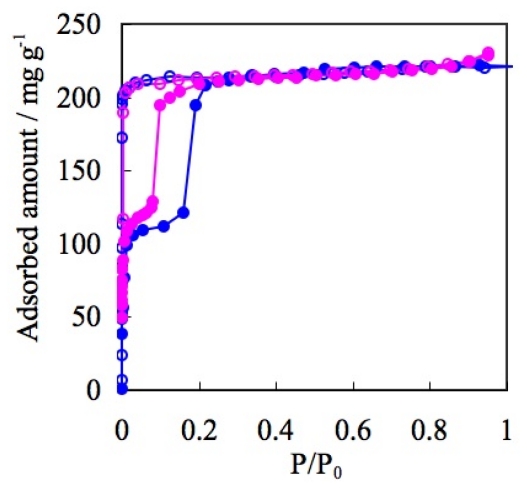
Adsorption isotherms of N_2_ on ELM-12 (Cu-OTf)(blue circles) and ELM-22 (Co-OTf)(pink circles) at 77 K. Solid and open symbols represent adsorption and desorption, respectively.

Another OTf containing ELM, ELM-22 ([Co(bpy)_2_(OTf)_2_]), accentuates the characteristics of OTf anions. Despite the differences of metal ions, both OTf containing ELMs (ELM-12 and ELM-22) indicate a similar adsorption property in the case of N_2_ at 77K [113]; the maximum amount of adsorption and definite double-step adsorption profile are close to each other with a slight difference in the gate pressure (Figure 12). These similar adsorption properties are apparently derived from the similarities in their fundamental structure. Counter anions regulate the structure (especially through the stacking mode) and influence the adsorption profile [114–120].

**Figure 13:**
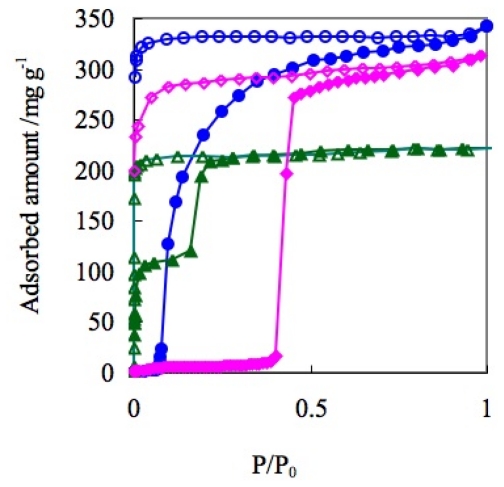
Adsorption isotherms of N_2_ on ELM-11 (Cu-BF_4_)(blue circles), ELM-12 (Cu-OTf)(green triangles), and ELM-13 (Cu-CF_3_BF_3_)(pink diamonds) at 77 K. Solid and open symbols represent adsorption and desorption, respectively.

Trifluoro(trifluoromethyl)borate anions, which contain a hydrophobic CF_3_ part like that in OTf anions, and a weakly coordinating BF_3_^−^ part that is the same as those of BF_4_^−^ which is also available for the construction of the ELM structures. ELM-13 was thynthesized by layering method (Cu(BF_4_) _2_ and KCF_3_BF_3_/H_2_O and bpy/acetone at room temperature) and the two dimensional layer strcture was analyzed by single crystal X-ray diffraction analysis. The pretreated (at 363 K under reduced pressure) CF_3_BF_3_^−^ containing ELM (ELM-13), [Cu(bpy)_2_(CF_3_BF_3_)_2_], shows unique gate gas adsorption properties. In the case of N_2_ adsorption at 77 K, ELM-13 shows a single step adsorption profile, while the maximum amount of adsorption is similar to BF_4_^−^ containing ELM-11. However, the vertical rising of the adsorption isotherm at the initial stage is more marked in OTf containing ELM-12 (Figure 13).

**Figure 14:**
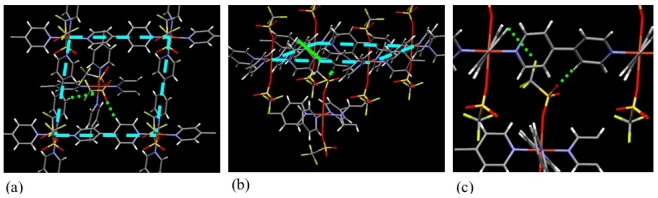
Hydrogen bonding between the counter anion and bpy of the neighboring layer (ELM-12): (**a**) square grid and OTf anions (top view), (**b**) square grid and OTf anions (side view), (**c**) hydrogen bonding between the OTf and the bpy.

In the case of ELMs, the role of counter anions is not only to charge compensation of metal cations, but also to regulate the interaction between the layers. In the case of ELM-12 (Cu-OTf), there exists the hydrogen bonding between the O atom of the OTf anion and the *β*-hydrogen atom of bpy in the neighboring layer, and the hydrogen bondings act as the tether line between the layers (Figure 14) [121].

The counter anions, BF_4_^−^ and CF_3_BF_3_^−^, have similar coordination properties (Metal-F) and both anions form the same type of hydrogen bonding (F...H). The two ELMs show a single step gate adsorption, and the maximum amount of adsorption (N_2_, 77 K) is almost similar. On the other hand, a double step gate-type ELM-12 contains OTf (CF_3_SO_3_^−^) anions, which resemble the CF_3_BF_3_^−^ anion in size, but differs in its coordination property (O...Met) and type of hydrogen bonding (S=O...H). In this context, it can therefore be presumed that the regulating factor of gate steps (single step or double steps) is not the size of the counter ion but their chemical factors, such as the coordination ability and type of hydrogen bonding. On the other hand, OTf and CF_3_BF_3_^−^ anion-containing ELMs almost show a vertical adsorption isotherm profile, while BF_4_^−^ containing ELM shows an upper convex profile. Therefore, in the case of the gate response, an important factor may be the ion size or the chemical property, such as hydrophobicity derived from a CF_3_ group. In any case, the ELMs, which have different counter ions but the same fundamental structure, show quite a strikingly different response to nitrogen gas, although nitrogen molecules are inert and small. Furthermore, it is also worth noting that the controlling factor for (1) gate behavior (single or double), (2) the gate profile (upper convex or vertical), and (3) the maximum amount of adsorption, are counter ions, which are just small parts attached to metal ions (Table 1).

**Figure 15:**
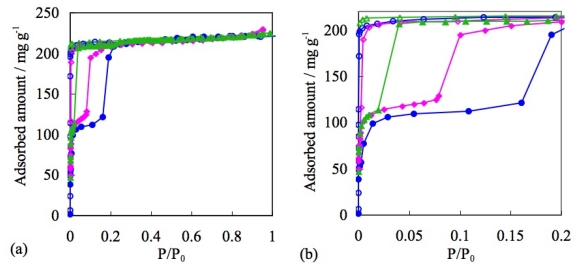
(**a**) Adsorption isotherms of N_2_ on ELM-12 (Cu-OTf) (blue circles), ELM-22 (Co-OTf) (pink diamonds), and ELM-12/3 (green triangles) (OTf/Cu-CF_3_BF_3_) at 77 K. (**b**) Close up of low-pressure region. Solid and open symbols represent adsorption and desorption, respectively.

As mentioned above, ELMs which contain OTf, BF_4_^−^, or CF_3_BF_3_^−^ anions, show characteristic adsorption isotherm profile, respectively. Then, what adsorption profile does an ELM, containing two different anions show [102,122,123]: ELM-12/3 bearing both OTf and CF_3_BF_3_^−^ was synthesized by layering of Cu(OTf)_2_ and KCF_3_BF_3_/H_2_O and bpy/ethanol (Elemental analysis as C_22_H_16_N_4_O_3_CuBF_9_S, which corresponds to the Cu:bpy:CF_3_SO_3_:CF_3_BF_3_ = 1:2:1:1; Calcd (%): C 39.93, H 2.44, N 8.47, Cu 9.60, B 1.63, S 4.85; found: C 41.4, H 2.30, N 8.8, Cu 9.60, B 1.20, S 4.40. The content of the two ions species are also quantitatively analyzed by ion chromatography: 1.04 equivalent of OTf for Cu^2+^ and 1.00 equivalent of CF_3_BF_3_^−^ for Cu^2+^ were detected.). Two dimensional layer strcture of ELM-12/3 was analyzed by single crystal X-ray diffraction analysis. This ELM-12/3 shows an adsorption isotherm quite similar to that of ELM-12 containing OTf: The similarities are shown in the vertical double step profile, the adsorption amount ratio of one-step and second-step (*ca.* 1/1), and the maximum amount of adsorption (220 mg/g). On the one hand, the gate pressure of ELM-12/3 decreases significantly compared to ELM-12 (OTf) and ELM-13 (CF_3_BF_3_^−^). On the other, ELM-12/3 shows the lowest gate pressure compared to ELM-12 (Cu-OTf) and ELM-22 (Co-OTf) (Figure 15).

To date, and to the best of our knowledge, ELM-12/3 is the only example of two-dimensional flexible CPs having the mixed counter anions.

### 3.2. Effect of Metal Cations

As mentioned above, the influence of metal ions on the adsorption profile is slight in the case of N_2_ adsorption at 77 K. If the counter ion is common, the slight difference of the adsorption isotherms between Cu-ELM *vs*. Ni-ELM and Cu-ELM *vs*. Co-ELM is observed only in the gate pressure. On the other hand, the kind of metal cations is quite an important factor for controlling the sorption phenomena in the case of O_2_ and CO_2_ adsorption [113]. Trifluoromethanesulfonate anion-containing ELM-12 (Cu) and ELM-22 (Co) are basically isostructures, despite the slight difference in the coordination field of metal ions derived from the Jahn-Teller effect. Therefore, the porous character of the initial structure derived from the slight zigzag stacking mode is common in both the ELMs. However, they show quite different responses to O_2_ and CO_2_ molecules. In the case of O_2_ adsorption at 77 K, the ELM-22 (Co) shows the same double step adsorption isotherms as those in the case of N_2_ adsorption, even though Cu^2+^ containing the ELM-12 adsorbs O_2_ in triple steps, and the maximum amount of adsorption is 1.6 times that of the ELM-22 (Co). Carbon dioxide molecules induce a similar response in the ELMs—the adsorption isotherms (196 K) of the ELM-22 (Co) are of a double step, while ELM-12 (Cu) shows a multi step adsorption phenomenon. Furthermore, the total amount of adsorption of ELM-12 (Cu) is 1.5 times that of ELM-22 (Co). The molecular number of that adsorbed on ELM-12 (Cu) and the physical properties of the adsorbed gas are shown in Table 2.

**Figure 16:**
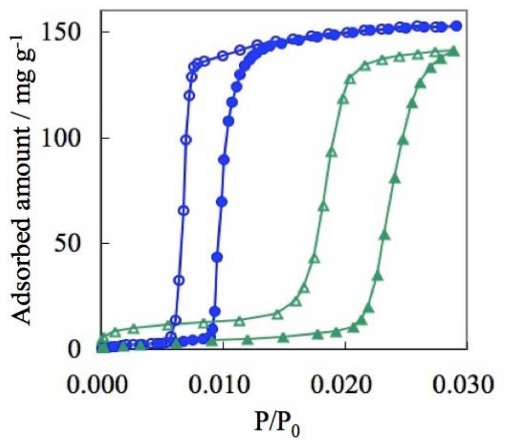
Adsorption isotherms of CO_2_ on ELM-11 (Cu-BF_4_)(blue circles) and ELM-31 (Ni-BF_4_)(green triangles) at 273 K. Solid and open symbols represent adsorption and desorption, respectively.

Although the molecular size of O_2_ is comparable to N_2_, the adsorbed amount of O_2_ is apparently larger compared to that of N_2_ (1.5 times). In addition, although the molecular size of CO_2_ is larger than N_2_, CO_2_ molecules of an amount similar to N_2_ can be accommodated in the flexible ELM-12 framework. Accordingly, it can be presumed that O_2_ and CO_2_ molecules have a stronger effect on the layer expansion of Cu-ELM and that such effect is less for Co-ELM. On the other hand, Ni^2+^ containing ELM-31 (Ni-BF_4_) shows simple one step gate responses to CO_2_ at 273 K, which is similar to the response of Cu^2+^ containing ELM-11 (Cu-BF_4_) to CO_2_ (Figure 16). In this case, the metal cation does not affect the gate profile but mainly the gate pressure.

It is also well known that the type of metal cation used has a strong effect on the adsorptivity of PCPs/MOFs [125–128]. This effect is divided into two categories: (1) direct effect—through the interaction between metal cations and adsorbed molecules, and (2) indirect effect—through the regulation of pore structures. In the case of the PCPs/MOFs having open metal sites, it is especially well studied that adsorption control by the metal ion is based on metal-adsorbate interaction [84,129–132]. Although it is also well known that isostructural PCPs/MOFs are constructed from different metal ions, the metal effects on adsorption through structural regulation have not been well studied, especially in the case of flexible coordination polymers [133–136]. Férey *et al*. report one of a few examples discussing the effect of large quadrupole moments of CO_2_ on the structural transformation of flexible PCPs/MOFs [135]. The contribution of the quadrupole moment of CO_2_ in the interaction is still much smaller than the dispersion attractive interaction. In the case of ELMs, an important aid with the large quadrupole moment of CO_2_ for large amount and multi step CO_2_ adsorption on ELM-12 (Cu) can be considered [135,137–140]. Carbon dioxide also shows specific behavior with the adsorption phenomena on the gate opening mechanism of ELM-11 (Cu-BF_4_). Although both N_2_ (77 K) and CO_2_ (273 K) show a single step gate adsorption on ELM-11, the number of adsorbed molecules is quite different: N_2_ is 7.0/unit cell (at P/P_0_ = 0.99, at 77 K) and CO_2_ is 1.9/unit cell (at P/P_0_ = 0.99, at 273 K), because the adsorption temperature of CO_2_ is close to the critical temperature as shown in Figures 13 and 16. Actually, the isosteric heat adsorption of CO_2_ on ELM-11 was estimated at 26 kJ/mol by the van’t Hoff equation, at the range of the number of adsorbed CO_2_ molecules from 0.5/unit cells (37.58 mg/g) to 1.5/unit cells (112.7 mg/g). This value is comparable to the sublimation enthalpy of CO_2_ (25 kJ/mol). These results imply the specific interaction of CO_2_ and ELM-11 at the subcritical temperature. Thus, CO_2_ should be highly stabilized in the lattice of ELM-11. Although Baiker *et al*. reported interaction between Cu^2+^ of (pre)ELM-11 and adsorbed acetnitrile molecule [141], metal-guest interaction of ELMs are still unclear and under investigation.

### 3.3. Hydrogen Adsorption

Hydrogen molecules are not adsorbed on nonporous ELM-11 under the condition of supercritical gas above 33 K. In addition, hydrogen molecules do not cause the structural transformation because of weak interaction. As both ELM-12 (Cu) and ELM-22 (Co), which contain OTf ions, are porous even at the initial stage, they adsorb slight H_2_ at 77 K. From the small amount of adsorbed gas and weak interaction with H_2_, the adsorption mechanism is considered as a quasi-micropore filling whereby super critical gas can be filled in the inherent pore sites enough to stabilize the molecules even above a critical temperature. The adsorption is not chemisorptive but reversible [142]. Although the pore parameters of ELM-12 (Cu) and ELM-22 (Co) are quite similar, the adsorbed H_2_ amounts differ from each other [113]. This difference is apparent especially in low pressure regions. For example, ELM-22 (Co) adsorbed more than 1.5 times that of adsorbed ELM-12 (Cu) at a low pressure region. As the difference in the dispersion interaction of Cu^2+^ and Co^2+^ ions with H_2_ molecules should be small, this difference is attributed to the more meandering pore structure of ELM-22 (Co) compared to ELM-12 (Cu), and the fine structural difference arises from a difference in metal cations (Table 3).

As mentioned above, in the case of ELMs, metal cations act not only as a simple connecting node for the architecture, but also as fine tuning of pore structures.

### 3.4. Effect of Ligand

In general, the length of ligands is a key factor to tune the coordination space of PCPs/MOFs [143–150]. Yaghi’s group reported the archetype study of relationships between the length of the ligand and the amount of gas adsorption using a series of various ligands [43]. According to their studies, PCP/MOF with longer ligands apparently tend to have a large coordination space—when the ligands are changed from terephthalic acid (IRMOF-1, contains one phenyl (Ph) ring), 4,4′-biphenldicarboxylic acid (IRMOF-10, two Ph ring), to 4,4′-terphenyldicarboxylic acid (IRMOF-16 three Ph ring), the calculated percentage of free volume increases from 79.2% (one Ph), 87.0 (two Ph), to 91.1% (three Ph).

In contrast to the Yaghi’s rigid PCPs/MOFs series, flexible ELMs show a reverse tendency. Although the extended ligand, 4,4′-bis(4-pyridyl)benzene (bpb) (11.4 Å) is 63% longer than bpy (7.0 Å) [151], ELM-31b ([Ni(bpb)_2_(BF_4_)_2_]) shows a 40% smaller adsorbed amount (W_0_(N_2_) = 212 mg/g at 77 K) compared to that of ELM-31 ([Ni(bpy)_2_(BF_4_)_2_])(W_0_(N_2_) = 350 mg/g at 77 K). This reverse tendency should be understood from the unique adsorption mechanism of ELMs. In the case of “hard” PCPs/MOFs, which show type I physisorption isotherms, there is a tendency for the larger free volume to accommodate more gas. On the other hand, “flexible” ELMs adsorb gas through clathrate formation; the adsorption depends on the stability of the gas-CP/MOF clathrates. Since the clathrates of larger square grids with longer ligands (bpb ligand, 15 × 15 Å) are unstable because of the weak interaction between guests and hosts, compared to small square grids (bpy ligand, 11 × 11 Å), the amount of adsorbed gas tends to decrease for the longer ligand system. Fujita *et al*. reported the example of flexible two-dimensional layer stacking-type PCP/MOF with extended ligands. This PCP/MOF varies its structure with solvent exchange [152]. To our best knowledge, the ELM-31b is the only example of two-dimensional stacking PCP/MOF with extended ligands, which can change its structure by gas molecules, which interact with host framework by weaker interaction.

## 4. Various Two-Dimensional Square Grid Stacking-Type (2DSG) CPs/MOFs

### 4.1. Two-Dimensional Square Grid Stacking-Type CPs/MOFs: Structure and Functions

An aromatic compound containing nitrogen such as pyridine is one of the most popular coordinative functional groups, and hence many CPs/MOFs have been synthesized using exobidentate ligands bearing two pyridyl groups, such as bpy [153,154]. There are quite a few examples of metal-organic square networks with linear bifunctional spacer ligands [155]. To synthesize such kinds of CPs/MOFs, various ligands have been used: short or long [156–161], rigid or flexible [162,163], linear or inflectional [164,165], with functional group(s) [159,166–170], rotaxane-type [171], chiral-type [172], and so on. An example of a typical linear, rigid, exobidentate ligand would be 4,4′-bipyridine. A number of two-dimensional square grid stacking-type CPs/MOFs (2DSG-CP/MOF) with this ligand has been reported [173–176]. Because of the neutral nature of bpy, 2DSG-CPs/MOFs necessarily contain counter anions to compensate the positive charges of metal ions, and these negatively charged counterparts increase the diversity of 2DSG-CPs/MOFs. Various 2DSG-CPs/MOFs constructed with bpy and unidentate coordination anion are listed in Table 4.

Since almost all the listed 2DSG-CPs/MOFs include guest molecules, this means the 2DSG-CPs/MOFs are potentially acting as porous materials. From the standpoint of host/guest chemistry, the synthesis of hybrid-type nonlinear optical materials was attempted with the combination of 2DSG-PCPs/MOFs host and guest molecules, such as *p*-nitroaniline [185]. On the other hand, there is no report on the gas adsorption or structural transformation of the CPs/MOFs listed in Table 4, except for ELMs. In addition, we cannot synthesize ELMs family with any counter anions other than BF_4_^−^, OTf, and CF_3_BF_3_^−^. If the scope of ligands widens from bpy to pyrazine, 1,4-bis(4-pyridyl)benzene, and 4,4′-bis(4-pyridyl)biphenyl, to the best of our knowledge, there is no report on the gate gas adsorption of 2DSG-CPs/MOFs or the structural transformation of 2DSG-CPs/MOFs caused by gas molecules. In the case of longer ligand, it is reported that the structural transformation of 2DSG-CPs/MOFs constructed with 4,4′-bis(4-pyridil)biphenyl ligand. However, the structural change was not caused by gas molecules but by the exchange of aromatic guest molecules [160]. The reason why all of the listed 2DSG-CPs/MOFs except the ELMs do not show the gas adsorption is considered to be as follows: (1) the cavity of the metal-organic square does not act as an open pore because of the close packing of the layers and the difficulty of structural transformation of the layers; (2) the cavity is already occupied by non-removable guest molecules and there is no space for gas adsorption; and (3) the 2DSG structure collapses when the guest is released. The necessary requirement for gas adsorption is conservation of the framework structure after the guest release. The collapse of the CP/MOF structure with the guest release can be seen as a common behavior, and in the case of 2D CP/MOFs [217], sometimes a turbostratic disorder occurs in the case of 2DSG CP/MOFs [218]. The present collapse assumption must be reconsidered, based on the fact that ELM-22 (Co) retains its 2DSG structure without any guest.

Hereafter, let us consider the role of counter anions. It is well known that these play a significant role in regulating the structure of CPs/MOFs [122,159,219–221]. In addition, the counter anions sometimes play a significant role in the function of CPs/MOFs. However, in the case of flexible PCPs/MOFs [222], the role of counter anions in structural transformation is not necessarily clear. The common features of the counter anions of ELMs are: (1) monodentate; (2) mono-valent; (3) weak coordination ability; (4) occupation of the apical positions; and (5) participation of fluorine atoms. In the case of non-ELM 2DSG-CPs/MOFs, NCS^−^, which have relatively strong interaction with metal cations, tend to occupy the apical positions. Imamoto reported the synthesis of [Ni(bpy)_2_(NCS)_2_] [188] of a fundamental structure is similar to ELM-22 (Co-OTf) having no guest and precise layer stacking. Therefore, the non-porous character of the Imamoto’s Ni-CP/MOF may be attributed not to structural friability but to the difficulty in the structural transformation for the generation of micropore. Jacobson reported the Co version of NCS-2DSG-CP/MOF, [Co(bpy)_2_(NCS)_2_]·2Et_2_O [179]. Although this compound easily releases two ether molecules, the non-guest state does not induce gas adsorption. In this case, the initial porous state is supposed to transform into a non-porous form (a compact layer stacking form) with the guest release. Imamoto and Jacobson did not mention the gas adsorption ability of their CPs/MOFs. From our adsorption experiment (N_2_ at 77 K and CO_2_ at 273 K, after the pretreatment at 363 K for three hours under reduced pressure), Ni-CPs/MOFs did not show any gas adsorption ability. We also checked Fujita’s [Cd(bpy)_2_(NO_3_)_2_], but this CP did not show N_2_ gas adsorption ability at 77 K.

In the case of other anions, such as NO_3_^−^, ClO_4_^−^, and PF_6_^−^, these weak coordination anions tend to locate themselves in the square grid as guests. They occupy the apical position only if the grid accommodates aromatic guest molecules. Based on these facts, occupation of the apical positions by weak coordinating anion can be regarded as one of the key factors for the gate gas adsorption phenomena. Although the space of the grid will decrease, if the counter anion is present as a guest, there still remains room for small gas molecules, considering the small size of the counter ions compared to the size of the square grid. Therefore, the reason why the ion-accommodating 2DSG-CP/MOF does not uptake the gas molecule, may be attributed to the difficulty of interlayer sliding. In the case of ELMs, interlayer sliding is a crucial motion for the gate phenomena. Therefore, the interlayer interaction is a relatively important factor for gate adsorption. In general, ligand-ligand interaction, such as CH-π and π-π, is well known as the interlayer interaction [223,224], and ligand-counter ion interaction also sometimes have a significant function [156,225–227]. In the case of ELMs, a counter ion occupies the apical positions of metal cations and acts as a terminal ligand. At the same time, the anion forms hydrogen bonds with the *β*-hydrogen of bpy of the neighboring layer, and the hydrogen bonding network acts as tether lines between the two-dimensional layers. These facts are strongly indicative of the key role of the counter anion in the gate phenomena.

The fact that the counter ions of ELMs (BF_4_^−^, CF_3_SO_3_^−^, and CF_3_BF_3_^−^) necessarily bear a number of fluorine atoms does not seem to be a coincidence. Although fluorine atoms bound to the carbon atom rarely form hydrogen bonding, fluorinated ligands sometimes affect the structural transformation or the adsorption phenomena of CPs/MOFs through their unique physical properties [228–234]. Fluorine atoms bound to inorganic elements have the ability to form hydrogen bonds, which sometimes play an important role in the construction of molecular structures. In addition, the largest electronegativity of the fluorine atom should influence the coordination space. However, the effect of fluorine atoms on the structural transformation of CPs/MOFs has not been systematically studied. As mentioned above, some sorts of participation of fluorine atoms on the gate phenomena can be presumed, and further study is required to clarify the accurate role of fluorine atoms.

### 4.2. Two-Dimensional Square Grid (2DSG) CPs/MOFs with Various Ligands Other Than bpy

As already mentioned, various 2DSG-CPs/MOFs which have a shorter ligand than bpy have been reported. Although some of them contain coordinating BF_4_^−^ or OTf, to the best of our knowledge, no gate gas adsorption phenomenon or structural transformation has been reported on these CPs/MOFs. There are a few reports on CPs/MOFs with longer analogues to bpy, such as 1,4-bis(4-pyridyl)benzene and 4,4′-bis(4-pyridyl)biphenyl [235]. Fujita *et al*. reported that the NO_3_^−^ coordinated 2DSG-CP/MOF with 4,4′-bis(4-pyridyl)biphenyl shows a reversible structural transformation, which was caused by guest exchange (mesitylene/*o*-dibromobenzene) with a very slow timescale in 22 hours [160]. To the best of our knowledge, this is the only report on the structural transformation of the isostructural CP/MOF of ELMs.

### 4.3. The Gate Phenomena of ELMs

In the case of ELMs, gate gas adsorption/desorption suddenly occurs, accompanied with the synchronous IR change [191]. From the isosbestic points of IR change, the structural transformation is understood as an equilibration between close and open forms. Strictly speaking, this phenomenon must be understood by clathrate formation through the guest molecule inclusion reaction, as already mentioned earlier [73,75,78,96]. The clathrate formation mechanism is described by a general thermodynamic expression. The application of this theory to ELM-11 is given in the following Equations (1) and (2):

(1) $$\mathrm{ELM}+n{\mathrm{CO}}_{2}\overset{K_{C}}{↔}\mathrm{ELM}-{\left({\mathrm{CO}}_{2}\right)}_{n}$$

(2) $$K_{C}=\frac{\left[\mathrm{ELM}-{\left({\mathrm{CO}}_{2}\right)}_{n}\right]}{[\mathrm{ELM}]P_{{\mathrm{CO}}_{2}}^{n}}$$

n shows the number of CO_2_ molecules accommodated in the unit cell of ELM-11 at the same time. In general, the cooperative structural transformation phenomena of oligomeric structures caused by plural effecters are well known as an allosteric effect (e.g., structural transformation of hemoglobin caused by O_2_ molecules). In the case of ELM-11, CO_2_ adsorption isotherms and the fitting results of the cooperative clathrate formation show relatively good accordance. This strongly indicates that the gate adsorption/desorption of ELM-11 stems from a clathrate formation reaction.

**Figure 17:**
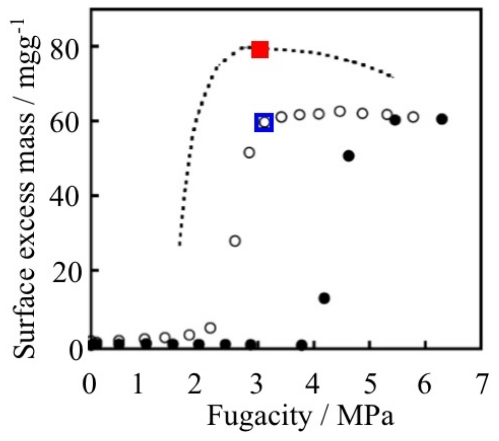
Temperature jump experiment of CH_4_ adsorption on ELM-11. The open and closed circle show CH_4_ adsorption and desorption on ELM-11 at 303 K, respectively. The temperature was dropped from the blue square (303 K) to the red square (273 K) and was then elevated from the red square to the blue square. The thin dashed line shows desorption branch at 273 K, which was measured by another experiment.

The gate adsorption/desorption phenomena of CPs/MOFs have been discussed both from the viewpoint of kinetics and thermodynamics [236–238]. In the case of ELMs, it is confirmed that both the gate opening and closing states are in a thermal equilibrium [96]. Methane gas-pressurized ELM-11 at 303 K at 2.5 MPa is in the gate-open stage. When the sample was cooled to 273 K, the adsorption amount increased and the amount was coincident with that of the desorption branch of 273 K. When the cooled sample was, in turn, warmed to 303 K, the adsorption amount was decreased and the amount was coincident with that of the desorption branch of 303 K (Figure 17). From these experiments, it is apparent that ELM-11 at the gate-open state is a thermodynamic product. The thermal equilibrium of the gate-closed state of ELM-11 was also confirmed by CO_2_ adsorption experiments. Although the gas pressure was retained for 13 hours at ambient temperature at slightly under the gate opening pressure, ELM-11 did not adsorb the gas. This experiment also strongly supports the equilibrium nature of ELM-11 before the gate opening.

As shown in Figure 14, the presence of hydrogen bonding between the counter ion and bpy ligand in neighboring layer is confirmed by single crystal X-ray diffraction analysis. During the early stage of the study of ELMs, gate phenomena were considered to accompany the cleavage of the hydrogen bonding network. Although the modulation of hydrogen bonding during the expansion/shrinkage structural transformation was not clarified, it is confirmed that interlayer interactions, such as hydrogen bonding and π-π interactions, still remain after the structural transformation. The weak interactions work as tether lines between the layers and the structural transformation can be achieved not by the cleavage of weak interactions but by the rotation of the aromatic rings and conformational change of counter ions [239–244]. The fact that the maximum adsorbed gas amount was determined by the nature of the counter ions suggests the presence of the tether line mechanism of hydrogen bonding after the structural transformation.

## 5. Evaluation of the Adsorptivity of ELM-11 from the Standpoint of Practical Application

### 5.1. Applicability of Gate Phenomena for the Energy-Saving Pressure Swing Adsorption Process

**Figure 18:**
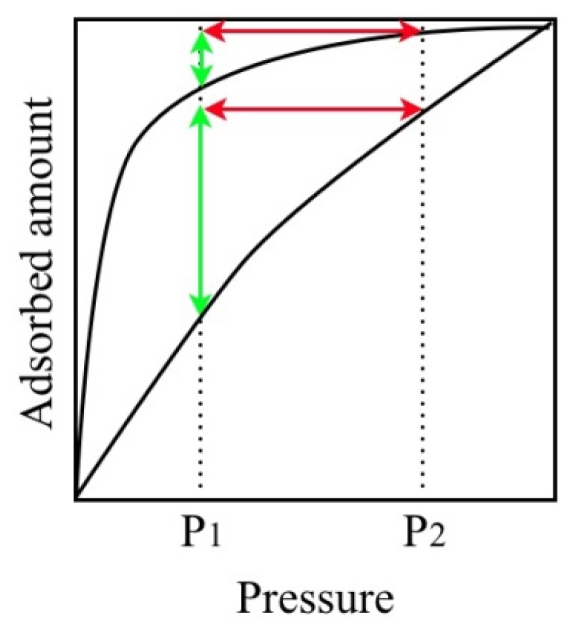
Representative example of two types of adsorption isotherm and the relationship between the pressure swing width and the amount of recoverable gas. The red arrow shows the pressure swing width and the green arrow shows the recovered gas amount by the pressure swing from P_2_ to P_1_, respectively.

Pressure swing or temperature swing adsorption systems using porous adsorbents have been practically used in gas separation, such as that for O_2_/N_2_ and CO_2_/CH_4_ [135,245,246]. In both systems, the maximum adsorption amount is an important factor for efficiency. However, the desorption amount per pressure or temperature swing is also important and this factor is strongly influenced by the profile of the adsorption isotherm (Figure 18). An adsorbent which shows a strong affinity to an adsorbate shows a steep uprise of adsorption isotherm at the low pressure region and such kinds of adsorbent sometimes have difficulty in recovering the adsorbed gas.

**Figure 19:**
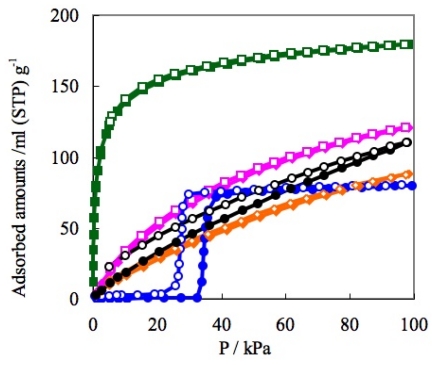
Carbon dioxide adsorption isotherms on various adsorbents at 273 K: green = zeolite 13X (13X APG); pink = Basolite™ C300 (HKUST-1); orange = Basolite™ A100 (MIL-53 (Al)); black = activated carbon fiber A-20; blue = ELM-11. Basolite™ C300 and Basolite™ A100 were purchased from Sigma-Aldrich Co. and were pretreated at 473 K, 3 h *in vacuo* before adsorption measurement. Zeolite 13X APG was purchased from Union Showa K.K. and was pretreated at 523 K, 3 h *in vacuo* before adsorption measurement.

Figure 19 and Table 5 show the CO_2_ adsorption isotherms on various adsorbent and recovered gas amounts by the pressure swing process calculated by the adsorption isotherms. The maximum amount of adsorbed gas on ELM-11 is moderate compared to other type I isotherm adsorbents. However, the recovered gas amount from ELM-11 by the pressure swing simulation is the largest because of the specific gate profile.

If an adsorbate has a strong affinity to CO_2_ molecules, type I adsorption isotherm is obtained, but the affinity is too strong for the easy release of adsorbed gas. In this way, simple type I profiles pose the dilemma of the combination of strong adsorption and easy desorption. On the other hand, living systems solve this dilemma; hemoglobin realizes the strong adsorption and easy desorption of oxygen by cooperative structural transformation and the sigmoidal oxygen association-dissociation curve [247–249]. As in the case of hemoglobin, the gate profile, which is achieved by cooperative structural transformation, has the possibility to overcome the dilemma of strong adsorption and easy desorption. Therefore, the gate adsorption material has excellent potential applicability for the pressure swing adsorption process.

### 5.2. Carbon Dioxide Gas Selectivity of ELM-11

Around an ambient temperature and pressure, CO_2_ opens the gate of ELM-11 but N_2_ and O_2_ cannot open it at ambient temperature and pressure, while the gate of ELM-11 is opened by N_2_ and O_2_ at a much lower temperature or higher pressure. Therefore, it is anticipated that high CO_2_ separation ability form N_2_ and O_2_ at ambient conditions using ELM-11. Actually, the highly pure CO_2_ (>99%) was obtained from ternary mixture gas (CO_2_:N_2_:O_2_ = 40:47:13 mol%) using ELM-11 by the simple temperature swing operation [191]. This result shows the advantage of flexible gate materials for efficient gas separation.

### 5.3. Adsorption Kinetics

**Figure 20:**
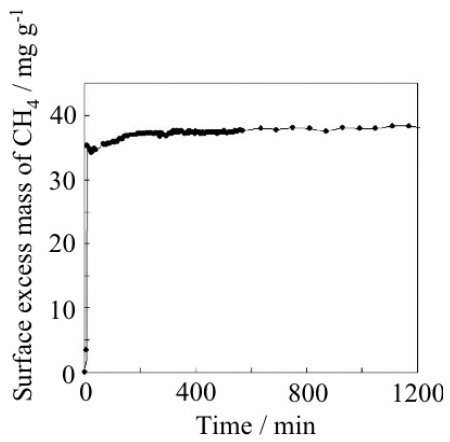
Adsorption speed of CH_4_ on ELM-11 at 6.0 MPa at 298 K.

The adsorption rate is an important factor for the industrial application of adsorbents. The adsorption rate of CO_2_ on ELM-11 was measured by the pressure jump method. In the case of the pressure jump from 150 to 735 Torr at 273 K and from 150 to 730 Torr at 298 K, half the amount of adsorption is reached within three minutes. Further, the adsorption rate of CH_4_ was also examined. When the pressure of CH_4_ increases from 2.0 to 5.5 MPa at 298 K, the adsorption amount peaks within one minute (Figure 20). From these experiments, it is revealed that the adsorption rate of ELM-11 is sufficiently rapid for practical use.

### 5.4. Molding of the Powder of ELM-11

**Figure 21:**
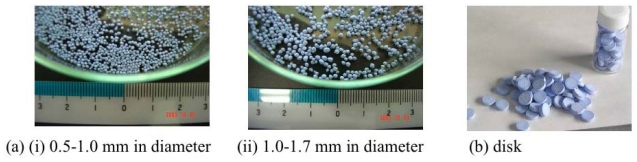
Shape forming of ELM-11: (**a**) pellet and (**b**) disk.

Powder adsorbents must be shape-formed for easy handling in the case of industrial application [250]. Therefore, the process for making ELM-11 pellets was studied. A certain amount of pellet samples (10 mm in diameter, 3 mm in thickness, ρ = 1.3g/cm^3^) were made using a continuous pressing pelletizer with magnesium stearate (10 wt%) as a lubricant. The granulation of ELM-11 was also studied. Relatively hard (bead hardness = 130 cN, 1 mm diameter, 20 beads) granules with narrow particle distribution were obtained by a commonly used carbon granulation process using sugar as a binder (Figure 21).

**Figure 22:**
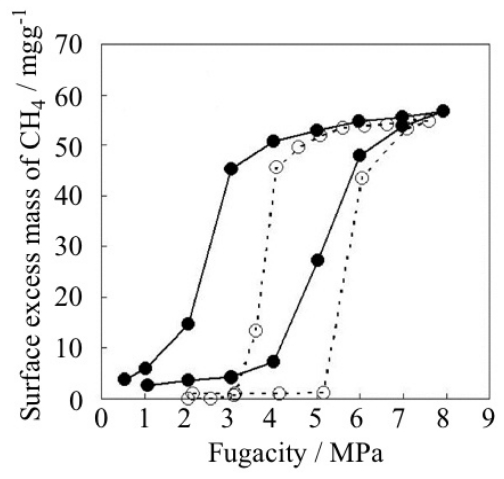
Effect of shape forming on the gas adsorption property at 298 K: Powder sample (dashed line, open circle) and pelletized sample (0.5–1.0 mm in diameter; solid line, closed circle).

The effect of the shape forming on the gas adsorptivity was estimated by the CH_4_ gas adsorption on granulated ELM-11. Although there was a slight decrease of gate adsorption/desorption pressure, it retained a definite gate profile and large hysteresis, and the maximum amount of CH_4_ adsorption was the same as that of unprocessed powder samples (Figure 22). Hence, easily obtainable ELM-11 powders are highly promising for real applications.

### 5.5. Temperature Elevation with the Gate Adsorption of CH_4_ on ELM-11

**Figure 23:**
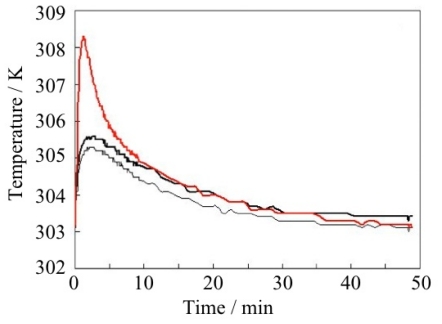
Rapid CH_4_ adsorption on ELM-11: The temperature was measured at the center of the sample (red line) and at the circumference (two positions: rigid line and thin line).

Methane gas is adsorbed on ELM-11 rapidly as mentioned above and the temperature elevation of ELM-11 with the adsorption of CH_4_ was examined. Fifty grams of preELM-11 was packed in a stainless steel column, and the column was heated to 393 K under reduced pressure to convert the preELM-11 to ELM-11. Next, CH_4_ was introduced to the column at 5.0 MPa, the pressure was increased from 5.0 to 6.0 MPa suddenly at 298 K, and the temperature of the adsorption column was monitored. The measured temperature elevation was only 3 K at the circumference of the column and only 6 K even at the center of the column (Figure 23). The maximum desorption due to the temperature rise is estimated to be less than 8%. If we mix the conductive carbon fibers with ELM-11 powder, the temperature rise should be suppressed.

### 5.6. Stability of ELM-11

ELM-11 is hygroscopic and varies its structure to the preELM-11 when exposed to the air. Therefore, it is convenient that ELM-11 is stored in the form of preELM-11. As mentioned above, preELM-11 is easily converted to ELM-11 by heat treatment at 393 K under reduced pressure. The precursor preELM-11 is quite stable and can be stored at room temperature. No structural and adsorption performance degradation was observed when stored as the form of preELM-11 for six years in a plastic vial at room temperature.

The stability of ELM-11 as an adsorbent was examined by repetition of CH_4_ adsorption-desorption experiments at 303 K. After the adsorption-desorption cycle was performed 50 times, almost no change in the maximum amount of adsorption and gate pressure (adsorption and desorption) was observed. It is noteworthy that the stacked layer architecture through weak interlayer interaction, such as hydrogen bonding and π-π interaction, shows such durability for the interlayer sliding and layer expansion/shrinkage modulation.

The heat stability of ELM-11 was examined by thermal gravimetry. The precursor preELM-11 released water molecules up to 420 K and changed its structure to ELM-11. No further weight loss was observed up to 420 K.

PreELM-11 is easily prepared according to the reported procedure [99] and commercially available from Tokyo Chemical Industry Co., Ltd. Accordingly, from both the stand points of properties and availability, ELM-11 could be applicable to an industrial separation process.

## 6. Catalytic Reaction of bpy Containing Two-Dimensional Layer PCPs/MOFs

Although there are a dozen reports on reactions catalyzed by PCPs/MOFs [35,38,251–257], there are only a few examples of catalytic reaction using bpy containing 2DSG PCPs/MOFs. Fujita *et al*. reported on the cyanosilylation of aldehyde catalyzed by 2DSG-PCP/MOF, [Cd(bpy)_2_(NO_3_)_2_] [204]. Arai *et al*. reports the catalytic oxidation of ketones, and Baiker *et al*. reports the catalytic epoxide ring opening reaction using [Cu(bpy)(H_2_O)_2_(BF_4_)_2_]·bpy (preELM-11) [258–260]. The catalyst is not 2DSG ELM-11 but the one dimensional-CP, preELM-11. But preELM-11 changes its structure in alcoholic solvent: Transformation from preELM-11 to ELM-11 by immersion in alcohol at room temperature was confirmed by IR, powder XRD, and gas adsorptivity. Baiker *et al*. indicate the dehydration of preELM-11 by methanol soaking [260]. Therefore there may be a possibility that the true catalytic species may be ELM-11. One of the most interesting points of ELM-11 catalysts is the effect of the flexibility on the reaction. Reports on the utilization of the flexible nature of PCPs/MOFs to improve the selectivity or reactivity of catalytic reactions will no doubt appear on the scene in the near future.

## 7. Conclusions

The rigid and linear exobidentate ligand, 4,4′-bipyridine (bpy) is one of the simplest ligands for the construction of PCPs/MOFs. The square grid structure constructed with bpy is one of the most fundamental motifs of the PCPs/MOFs structure. The two-dimensional square-grid layer stacking (2DSG) PCPs/MOFs, with appropriate metal cations and counter anions, only show structural transformation with the gate gas adsorption phenomena and such special 2DSG-PCPs/MOFs are called “elastic layer-structured metal-organic frameworks (ELMs)”. In this review, we show a brief survey of general 2DSG-PCPs/MOFs with bpy and also survey the structure and function of the ELMs based on study of the author’s group.

Since the gate profile can put the desorption pressure close to adsorption pressure, these unique phenomena can make it possible to overcome the dilemma of strong adsorption and easy desorption, which is one of the ideal properties for a practical adsorbent. The fact that such a unique property can be achieved through the simple 2DSG-structure and the gate property can be regulated by changing the metal cation and counter ion while retaining the fundamental structure, is attractive from both academic and industrial perspectives.

The ultimate functional organic architecture is animate beings. They have developed dexterous biological flexible structures using weak interactions. DNA and RNA store the genetic information in the hydrogen bonding between the base pairs and the enzyme’s flexible nature derived from weak bonding improves the catalytic ability [261]. The “flexible adsorbent”, hemoglobin attain the easy-adsorption and easy-desorption of oxygen. “Flexibility” should be the key factor which makes up the PCPs/MOFs as some of the ultimate artificial functional materials.

## Figures and Tables

**Table 1:** Counter ions and adsorption properties of ELMs.

ELM-	Counter Ion	Coordination Bond	Hydrogen Bond	Gate Type	Amount of Gas Adsorption^a^/mg g^−1^
11	BF_4_^−^	F-Met	F-H	one step	340
12	OTf	O-Met	S=O-H	two steps	220
13	CF_3_BF_3_^−^	F-Met	F-H	one step	314

a Nitrogen adsorption at 77 K.

**Table 2:** Adsorbed molecular numbers per one copper atom of ELM-12 and parameters of gas molecules a,b.

Parameters	N_2_	O_2_	CO_2_
Adsorbed molecular numbers per one copper atom of ELM-12	5.3	7.8	5.6
Quadrupole moment (10^−40^ Cm^2^)	−4.9	−1.33	−14.9
Lennard-Jones potential (e/k_B_/K)	104.2	126.3	245.3

a Nitrogen and O_2_ adsorption was measured at 77 K and CO_2_ was at 196K.

b References for the parameters: [124,129,137,138].

**Table 3:** Adsorption amount of H_2_ and pore parameters^a^ of ELM-12 (Cu-OTf) and ELM-22 (Co-OTf).

	ELM-12 (Cu-OTf)	ELM-22 (Co-OTf)
H_2_ adsorption amount [mg g^−1^, 1 atm at 77 K]	5.9	6.8
surface area [m^2^ g^−1^]	390	400
micropore volume [mL g^−1^]	0.14	0.15
adsorption capacity [mg g^−1^]	118	125
total pore volume [mL g^−1^]	0.27	0.28
isosteric heat of adsorption [kJ mol^−1^]	12.2	13.0

a All pore parameters were estimated from N_2_ adsorption isotherms measured at 77 K by using Dubinin-Radushkevich equation and/or liquid nitrogen density.

**Table 4:** Two-dimensional square grid stacking type CPs/MOFs constructed with bpy and unidentate coordination anion.

Compound^a^	Function^b^	Apical Ligand	Counter Ion	References
[M(bpy)_2_(dtbp)_2_]·2H_2_O (M = Mn, Co, Cd)	GI	H_2_O	dtbp	[177,178]
[Co(bpy)_2_(NCS)_2_]·2Et_2_O	GI	NCS^−^	NCS^−^	[179]
[Co(bpy)_2_(H_2_O)_2_]·3NTf_2_·mim	GI	H_2_O	NTf_2_	[180]
[Co(bpy)_2_(H_2_O)_2_]·2ps·10H_2_O	GI	H_2_O	ps	[181]
[Co(bpy)_2_(H_2_O)_2_]·2NO_3_·2bpy·2H_2_O	GI	H_2_O	NO_3_^−^	[182]
[Co(bpy)_2_(H_2_O)_2_]·bpy·bsb	GI	H_2_O	bsb	[183]
[Co(bpy)_2_(OTf)_2_] (ELM-22)	GA	OTf	OTf	[113]
[M(bpy)_2_(NO_3_)_2_]·3np (M = Co, Ni)	GI	H_2_O	NO_3_^−^	[184]
[M(bpy)_2_(NO_3_)_2_](na)_2_ (M = Co, Ni, Zn)	GI	NO_3_^−^	NO_3_^−^	[185]
[M(bpy)_2_(NO_3_)_2_]·arenes (M = Co, Ni)	GI	NO_3_^−^	NO_3_^−^	[186]
[Ni(bpy)_2_(BF_4_)_2_] (ELM-31)	GA	BF_4_^−^	BF_4_^−^	[113]
[Ni(bpy)_2_(NO_3_)_2_]·2pyrene	GI	NO_3_^−^	NO_3_^−^	[187]
[Ni(bpy)_2_(NCS)_2_]	-	NCS^−^	NCS^−^	[188,189]
[Ni(bpy)_2_(H_2_PO_4_)_2_]·G G = *n*-BuOH·H_2_O, 2bpy·3H_2_O, or 2bpy·ethylene glycol·H_2_O	GI	H_2_PO_4_^−^	H_2_PO_4_^−^	[190]
[Cu(bpy)_2_(BF_4_)_2_] (ELM-11)	GA	BF_4_^−^	BF_4_^−^	[99]
[Cu(bpy)_2_(OTf)_2_] (ELM-12)	GA	OTf	OTf	[67]
[Cu(bpy)_2_(CF_3_BF_3_)_2_] (ELM-13)	GA	CF_3_BF_3_^−^	CF_3_BF_3_^−^	[99]
[Cu(bpy)_2_(OTf)(CF_3_BF_3_)] (ELM-12/3)	GA	CF_3_BF_3_^−^, OTf	CF_3_BF_3_^−^OTf	[191]
[Cu(bpy)_2_(H_2_O)_2_]·2ClO_4_·bpo·3H_2_O	GI	H_2_O	ClO_4_^−^	[192]
[Cu(bpy)_2_(H_2_O)_2_]·2ClO_4_·H_2_O	GI	H_2_O	ClO_4_^−^	[193]
[Cu(bpy)_2_(H_2_O)]·2sac·CH_2_Cl_2_	GI	H_2_O	sac	[194]
[Cu(PF_6_)(bpy)_2_(CH_3_CN)]·PF_6_·2CH_3_CN	GI	PF_6_^−^, CH_3_CN	PF_6_^−^	[122]
[Cu(bpy)_2_(H_2_O)_2_]·PF_6_·BF_4_	GI	H_2_O	PF_6_^−^·BF_4_^−^	[122]
[Cu(bpy)_2_(H_2_O)_2_]·2PF_6_	GI	H_2_O	PF_6_^−^	[122]
[Cu(bpy)_2_(H_2_O)_2_]·(UO_2_·Hcit)_2_·7H_2_O	GI	H_2_O	UO_2_·Hcit	[195]
[Cu(bpy)_2_(H_2_O)_2_]·2sac·DMF	GI	H_2_O	sac	[196]
[Cu(bpy)_2_(H_2_O)_2_]·2PF_6_·2H_2_O·2tdp	GI	H_2_O	PF_6_^−^	[197]
[Cu(bpy)_2_(H_2_O)_2_]·4ClO_4_·H_2_bpy	GI	H_2_O	ClO_4_^−^	[198]
[M(bpy)_2_(H_2_O)_2_]·2ClO_4_·(2,4′-bpy)_2_·H_2_O (M = Zn, Cd)	GI	H_2_O	ClO_4_^−^	[198]
[Cu(bpy)_2_(NO_3_)_2_]·3paba	GI	NO_3_^−^	NO_3_^−^	[199]
[Cd(bpy)_2_(H_2_O)_2_]·2NO_3_·4H_2_O	GI	H_2_O	NO_3_^−^	[199]
[Zn(bpy)_2_(H_2_O)_2_]·bpy·bs	GI	H_2_O	bs	[200]
[Zn(bpy)_2_(fcph)_2_]	ME	fcph	fcph	[201]
[Zn(bpy)_2_(NO_3_)_2_]·2dcb·pyrene	GI	NO_3_^−^	NO_3_^−^	[202]
[Cd(bpy)_2_]·2NO_3_	Cat	-	NO_3_^−^	[203]
[Cd(bpy)_2_(NO_3_)_2_]·2dbb	GI	NO_3_^−^	NO_3_^−^	[203]
[Cd(bpy)_2_(H_2_O)_2_]·2NO_3_·4H_2_O	Cat, GI	H_2_O	NO_3_^−^	[204]
[Cd(bpy)_2_]·2NO_3_·2dbb	GI	-	NO_3_^−^	[205]
[Cd(bpy)_2_(H_2_O)_2_]·2NO_3_·4H_2_O	GI	H_2_O	NO_3_^−^	[206]
[Cd(bpy)_2_(NO_3_)(H_2_O)]·NO_3_·2abp	GI	H_2_O, NO_3_^−^	NO_3_^−^	[206]
[Cd(bpy)_2_(NO_3_)_2_]·2na	GI	NO_3_^−^	NO_3_^−^	[207]
[Cd(bpy)_2_(H_2_O)_2_]·2ClO_4_·1.5bpy·cnp·4H_2_O	GI	H_2_O	ClO_4_^−^	[208]
[Cd(bpy)_2_(H_2_O)_2_]·bpy·2nan·2ClO_4_·H_2_O	GI	H_2_O	ClO_4_^−^	[209]
[Cd(bpy)_2_(ClO_4_)_2_]·2mna	GI	ClO_4_^−^	ClO_4_^−^	[209]
[Cd(bpy)_2_(H_2_O)_2_]·2PF_6_·2bpy·4H_2_O	GI	H_2_O	PF_6_^−^	[210]
[Cd(bpy)_2_(H_2_O)(OH)]·PF_6_	GI	H_2_O, OH^−^	PF_6_^−^, OH^−^	[210]
[Cd(bpy)_2_(H_2_O)_2_]·2BF_4_·2bpy·nab·2H_2_O	GI	H_2_O	BF_4_^−^	[211]
[Cd(ans)_2_(bpy)_2_]	GI	ans	ans	[212]
[Cd(bpy)_2_(H_2_O)_2_]·2pic	GI	H_2_O	pic	[213]
[Zn(bpy)_2_(H_2_O)_2_]·2pic·2H_2_O	GI	H_2_O	pic	[214]
[Zn(bpy)_2_(H_2_O)_2_]·bpy·2pic·H_2_O	GI	H_2_O	pic	[215]
[Cd(bpy)_2_(NO_3_)_2_]·G; G = chlorobenzene, dbb, or *p*-chlorobenzene	GI	NO_3_^−^	NO_3_^−^	[216]

a Abbreviations: Et_2_O = diethylether; dtbp = di-*tert*-butyl phosphate; mim = 1-butyl-3- methylimidazolium; NTf = bis(trifluoromethanesulfonyl)imide; ps = pyridine-4-sulfonic acid; bsb = 4,4′-bis(sulfonatostiryl)biphenyl; np = naphthalene; na = *p*-nitroaniline; arenes =chlorobenzene, *o*-dichlorobenzene, benzene, nitrobenzene, toluene, or anisole; bpo = 2,5-bis(3-pyridyl)-1,3,4-oxadiazole; sac = *o*-sulfobenzimidate; UO_2_·Hcit = uranyl citrate; tdp = 1,3,4-thiadiazole-2,5-di-4-pyridyl; paba = 4-aminobenzic acid; bs = benzenesulfonate; FcphSO_3_ = *m*-ferrocenyl benzenesulfonate; dcb = *o*-dichlorobenzene; dbb = *o*-dibromobenzen; abp = 4-amino-benezopheone; na = 2-nitroaniline; cnp = 4-chloro-2-nitrophenol; nan = *o*-nitroaniline; mna = *N*-methyl-2-nitroaniline; nab = *o*-nitroaminobenzene; ans = 2-aminonaphthalene-1-sulfonate; pic = picrate

b GI = guest inclusion, GA = gate adsorption, ME = metal ion exchange; Cat = catalysis.

**Table 5:** Maximum amount of adsorbed CO_2_ on various adsorbents and recovered gas amount by pressure swing at 273 K.

Adsorbent	Maximum amount of adsorbed gas/ml g^−1^^a^	Recovered gas amount by pressure swing (45→20 kPa)/ml g^−1^^b^
ELM-11	80	71
C300	121	30
A100	88	22
13X APG	179	13
A20	110	17

a Measured by BELSORP-miniII (BELL Japan INC).

b Calculated from the isotherms.
